# Tracking Control of a Leg Rehabilitation Machine Driven by Pneumatic Artificial Muscles Using Composite Fuzzy Theory

**DOI:** 10.1155/2014/464276

**Published:** 2014-03-18

**Authors:** Ming-Kun Chang

**Affiliations:** Department of Mechanical and Computer-Aided Engineering, St. John's University, No. 499, Section 4, Tam King Road, Tamsui District, New Taipei City 25135, Taiwan

## Abstract

It is difficult to achieve excellent tracking performance for a two-joint leg rehabilitation machine driven by pneumatic artificial muscles (PAMs) because the system has a coupling effect, highly nonlinear and time-varying behavior associated with gas compression, and the nonlinear elasticity of bladder containers. This paper therefore proposes a T-S fuzzy theory with supervisory control in order to overcome the above problems. The T-S fuzzy theory decomposes the model of a nonlinear system into a set of linear subsystems. In this manner, the controller in the T-S fuzzy model is able to use simple linear control techniques to provide a systematic framework for the design of a state feedback controller. Then the LMI Toolbox of MATLAB can be employed to solve linear matrix inequalities (LMIs) in order to determine controller gains based on the Lyapunov direct method. Moreover, the supervisory control can overcome the coupling effect for a leg rehabilitation machine. Experimental results show that the proposed controller can achieve excellent tracking performance, and guarantee robustness to system parameter uncertainties.

## 1. Introduction

In cases of traumatic brain injury, bone injury, amputation, or spinal cord injury caused by misfortunes such as traffic accidents and cerebral apoplexy, lower limb rehabilitation machine can help patients recover extremity functions by means of continuous passive motion (CPM). Traditionally, physical therapy for achieving functional rehabilitation is carried out by medical therapists on a person-to-person basis. However, recently many automatic rehabilitation devices have been gradually applied in physical therapy programs. Rehabilitation machines are usually driven by electric motors, which are typically rigid in nature. Because of this, actuators can generate discomfort or pain when interfacing with humans. For this reason, current electromechanical actuation systems should be replaced to ensure adaptability, conformity, and safety. An adequate actuator for a rehabilitation device must provide physically adjustable compliance and safety and ensure soft contact with the patient, similar to the behavior of human muscles. It has been suggested that pneumatic artificial muscles (PAMs) can contribute towards achieving more comfortable devices for interfacing with human limb segments.

PAMs behave in a manner very similar to the muscles that move the skeletons of animals and have many advantages, such as high power to weight ratio [1], high power to volume ratio [2], low maintenance, negligible mechanical wear, low cost, cleanliness, high reliability, flexibility, and compliance for use with humans. For these reasons, PAMs are commonly employed in rehabilitation engineering, nursing, and human-friendly therapeutic machine.

However, PAMs exhibit highly nonlinear and time-varying behavior due to the compression of air and the nonlinear elasticity of bladder containers. This makes it difficult for classical controllers to achieve excellent control performance. In recent years, researchers have developed a wide variety of approaches to overcome these problems. Noritsugu and Tanaka [3] developed four modes of linear motion with impedance control to control force during movement and used an adaptive identification method to estimate the system model. Lilly and Yang [4] applied a sliding mode controller to a planar arm actuated by two PMA groups; simulation results were consistent with theoretical findings for two different masses. Ahn and Anh [5] adopted an ARNN controller in a PAM manipulator for reducing tracking errors. Shen [6] developed a full nonlinear model that encompassed all the major existing nonlinearities. Based on this model, the standard sliding mode control approach was applied to obtain robust control, even in the event of model uncertainties and disturbances.

Since the inception of fuzzy set theory by Zadeh [7] in 1965, a great deal of research has been focused on fuzzy control systems. Takagi and Sugeno [8] proposed the T-S fuzzy model-based controller in 1985, and the T-S fuzzy model-based system subsequently emerged as one of the most active and fruitful areas of fuzzy control. Using a T-S fuzzy model-based controller, a complex dynamic model can be decomposed into a set of local linear subsystems via fuzzy inference. Stability analysis is carried out using the Lyapunov direct method, where the control problem is formulated into linear matrix inequalities (LMIs). Based on this approach, Ahn and Anh [9] also developed an inverse double nonlinear autoregressive model with exogenous control based on the T-S fuzzy model applied in a PAM robot. A novel *H*_*∞*_ control structure based on a Takagi-Sugeno model [10] was proposed to track the desired trajectories, and simulation results illustrated the efficiency of the proposed approach for the new rehabilitation device.

The leg rehabilitation machine driven by PAMs is a two-input, two-output system. This paper proposes composite fuzzy theory, which includes T-S fuzzy tracking control and supervisor control in order to improve tracking performance. The proposed approach decomposes the model of a nonlinear system into a set of linear subsystems with associated nonlinear weighting functions, enabling the use of simple linear control techniques without the need for complicated nonlinear control strategies, and also provides a systematic framework for the design of a state feedback controller [11]. It has been shown that a composite fuzzy control system can be guaranteed to be asymptotically stable if a common positive definite solution exists for a set of Lyapunov inequalities. In addition, the supervisory control can overcome the coupling effect due to two-joint motion. In view of the above advantages, the proposed controller was applied to the output tracking control of this system, and experimental results verified that the proposed controller is capable of achieving excellent tracking performance.

The remainder of the paper is organized as follows.  Section 2 describes the control strategies.  Section 3 describes the system. In Section 4, the dynamics of the model are derived. Experimental results for output tracking are shown in Section 5. Finally, conclusions are presented in Section 6.

## 2. Control Strategies

### 2.1. Takagi-Sugeno Fuzzy Tracking Controller

Consider a general nonlinear dynamic equation

(1) $$\begin{array}{c} \dot{x}\left(t\right)=f\left(x\left(t\right)\right)+g\left(x\left(t\right)\right)u\left(t\right)\\ y\left(t\right)=q\left(x\left(t\right)\right), \end{array}$$

where *x* ∈ *R* ^*n*^ is the state vector, *y* ∈ *R* ^*m*^ is the controlled output, *u* ∈ *R* ^*m*^ is the control input vector, and *f*(*x*), *g*(*x*), and *q*(*x*) are nonlinear functions with appropriate dimensions. The nonlinear system (1) can then be expressed by the fuzzy system.

Rule *i*:

(2) IF  z1(t)  is  F1i  and⋯and  zg(t)  is  FgiTHEN  x˙(t)=Aix(t)+Biu(t), i=1,2,…,r,

where *z*(*t*)_1_ ~ *z*_*g*_(*t*) are the premise variables including system states, *F*_*ij*_ denotes the fuzzy sets, *r* is the number of fuzzy rules, and *A*_*i*_ and *B*_*i*_ are system matrices with appropriate dimensions. For simplicity, this study assumed that the membership functions had been normalized; that is, ∑_*i*=1_^*r*^Π_*j*=1_^*g*^ *F*_*ji*_(*z*_*j*_) = 1. As in (1), using the singleton fuzzier, product inferred, and weighted defuzzier, the fuzzy system is inferred as

(3) $$\begin{array}{c} \dot{x}\left(t\right)=\sum_{i=1}^{r}h_{i}\left[A_{i}x\left(t\right)+B_{i}u\left(t\right)\right], \end{array}$$

where *h*_*i*_(*z*(*t*)) = Π_*j*=1_^*g*^ *F*_*ji*_(*z*_*j*_(*t*)). Note that ∑_*i*=1_^*r*^ *h*_*i*_(*z*(*t*)) = 1 for all *t*, where ∑_*i*=1_^*r*^ *h*_*i*_(*z*(*t*)) ≥ 0 for *i* = 1,2,…, *r* are regarded as grade functions.

For output tracking control, the control objective is required to satisfy

(4) $$\begin{array}{c} \overset{-}{y}\left(t\right)-r\left(t\right)⟶0\;\text{as}\;\;t⟶\infty , \end{array}$$

where *r*(*t*) denotes the desired trajectory or reference signal. To convert the output tracking problem into a stabilization problem, a set of virtual desired variables *x*_*d*_(*t*) was introduced, to be tracked by the state variable *x*. Let $\tilde{x}\left(t\right)=x\left(t\right)-x_{d}\left(t\right)$ denote the tracking error for the state variables. The time derivative of $\tilde{x}(t)$ yields

(5) $$\begin{array}{c} \dot{\tilde{x}}\left(t\right)=\dot{x}-{\dot{x}}_{d}=\sum_{i}^{r}h_{i}\left[A_{i}x\left(t\right)+B_{i}u\left(t\right)\right]-{\dot{x}}_{d}\left(t\right). \end{array}$$

If the control input *u*(*t*) is assumed to satisfy the following equation:

(6) $$\begin{array}{c} \sum_{i=1}^{r}h_{i}B_{i}\tau \left(t\right)=\sum_{i=1}^{r}h_{i}B_{i}u\left(t\right)+\sum_{i=1}^{r}h_{i}A_{i}x_{d}\left(t\right)-{\dot{x}}_{d}\left(t\right), \end{array}$$

where *τ*(*t*) is a new control to be designed, then the tracking error system (5) results in the following form:

(7) $$\begin{array}{c} \dot{\tilde{x}}\left(t\right)=\sum_{i=1}^{r}h_{i}A_{i}\tilde{x}\left(t\right)+\sum_{i=1}^{r}h_{i}B_{i}\tau \left(t\right). \end{array}$$

The design of the new control *τ*(*t*) is similar to solving a stabilization problem. The purpose is to steer $\tilde{x}(t)$ to zero, which means that state *x*(*t*) tracks *x*_*d*_(*t*). The new fuzzy controller *τ*(*t*) is designed on the basis of parallel distributed compensation (PDC) and is represented as follows:

Rule *i*:

(8) IF  z1(t)  is  F1i  and⋯and  zg(t)  is  Fgi:THEN  τ(t)=−Kix~(t),

where *K*_*i*_ represents feedback gain. The inferred output of the PDC controller is expressed in the following form:

(9) $$\begin{array}{c} \tau \left(t\right)=-\sum_{i=1}^{r}h_{i}K_{i}\tilde{x}\left(t\right). \end{array}$$

Substituting (9) into (7) yields

(10) $$\begin{array}{c} \dot{\tilde{x}}\left(t\right)=\sum_{i=1}^{r}\;\sum_{j=1}^{r}h_{i}h_{j}\left(A_{i}-B_{i}K_{j}\right)\tilde{x}\left(t\right). \end{array}$$

The stability analysis of this tracking system (10) is carried out using the Lyapunov direct method, and the Lyapunov function is defined as

(11) $$\begin{array}{c} V\left(\tilde{x}\left(t\right)\right)={\tilde{x}}^{T}\left(t\right)P\tilde{x}\left(t\right)>0, \end{array}$$

where *P* is a positive symmetric matrix. Taking the derivative of *V* with respect to time yields

(12) V˙(x~(t))=x~˙T(t)Px~+x~T(t)Px~˙(t)=∑i=1r ∑j=1rhihj(x~T(AiT−KjTBiT)Px~)+∑i=1r ∑j=1rhihjx~TP(Ai−BiKj)x~=∑i=1r∑j=1rhihjx~T[(Ai−BiKi)TP+P(Ai−BiKi)]x~.

The controller is stable if $\dot{V}<0$. Hence, the LMI form is expressed as follows:

(13) $$\begin{array}{c} {\left(A_{i}-B_{i}K_{i}\right)}^{T}P+P\left(A_{i}-B_{i}K_{i}\right)<0\;\text{for}\;i=1,2,\ldots ,r,\\ G_{ij}P+PG_{ij}<0\;1\leq i<j\leq r, \end{array}$$

where *G*_*ij*_ = (*A*_*i*_ − *B*_*i*_ *K*_*j*_ + *A*_*j*_ − *B*_*j*_ *K*_*i*_)/2 and *G*_*ii*_ = *A*_*i*_ − *B*_*i*_ *K*_*i*_.

The controller gain *K*_*i*_ is obtained using the LMI toolbox of MATLAB. If there exists a common positive definite matrix *P* that satisfies inequalities (13), it can be guaranteed that the tracking error will approach zero.

### 2.2. Composite Fuzzy Tracking Controller

Because the leg rehabilitation machine has a coupling effect due to mechanism interaction, many fuzzy model controllers in the related literature exhibit restrictive tracking control in application. The proposed approach introduces supervisory control in order to overcome the coupling effect. The *i*th rule of the proposed controller is defined as follows.

Rule *i*:

(14) IF  z1(t)  is  F1i  and⋯and  zg(t)  is  FgiTHEN  u(t)=Kix~(t)+us i=1,2,…,r,

where *u*_*s*_(*t*) ∈ *R* ^*m*^. The proposed controller consists of a local state feedback $K_{i}\tilde{x}$ and a supervisory control *u*_*s*_. Therefore, the output of the proposed controller is

(15) $$\begin{array}{c} u\left(t\right)=\sum_{i}^{r}h_{i}K_{i}\tilde{x}\left(t\right)+u_{s}. \end{array}$$

The closed-loop system is given by

(16) x˙(t)=∑i=1r ∑j=1rhihj[Ai−BiKj]x~(t)+∑i=1rhiBius(t)=∑i=1rhi2Giix~(t)+2∑i<jrhihjGijx~(t)+Bus(t).

Suppose that there exist a symmetric and positive definite matrix *P* and some matrices *K*_*i*_ so that the following reduced stability condition holds:

(17) $$\begin{array}{c} {\left(A_{i}-B_{i}K_{i}\right)}^{T}P+P\left(A_{i}-B_{i}K_{i}\right)\leq -Q_{i},\;i=1,\ldots ,r, \end{array}$$

where *Q*_*i*_ is a positive definite matrix. Based on this assumption, each subsystem is locally controllable, and a stable feedback gain is obtainable. Intuitively, a common matrix *P* that satisfies (17) can be obtained more easily than can one that fulfills the basic stabilization conditions. When the LMI method is applied, conditions (17) can be efficiently verified. If a feasible solution is obtained, the design proceeds to exploit the supervisory control in order to deal with the coupling effects.

Choose the Lyapunov function candidate, $V_{1}(x)={\tilde{x}}^{T}P\tilde{x}$. The time derivative of *V*_1_(*x*) is as follows:

(18) V˙1(x)=∑i=1rhi2x~T(GiiTP+PGii)x~+2∑i<jrhihjx~T(GijTP+PGij)x~+2x~TPBus≤−∑i=1rhi2x~TQix~+2∑i<jrhihjx~T(GijTP+PGij)x~+2x~TPBus.

Given the matrix property, clearly,

(19) λmin⁡(GijTP+PGij)||x~||2≤x~T(GijTP+PGij)x~≤λmin⁡(GijTP+PGij)||x~||2,

where *λ*_min⁡⁡(max⁡)_ denotes the smallest (largest) eigenvalue of the matrix. Define

(20) $$\begin{array}{c} \alpha =\underset{i,j}{\max}\lambda_{\max}\left(G_{ij}^{T}P+PG_{ij}\right)\;\text{for}\;1\leq i<j\leq r. \end{array}$$

A relaxed condition concerning the coupling effect is expressed as

(21) $$\begin{array}{c} \sum_{i<j}^{r}h_{i}h_{j}{\tilde{x}}^{T}\left(G_{ij}^{T}P+PG_{ij}\right)\tilde{x}\leq k_{1}{||\tilde{x}||}^{2},\;\;k_{1}=\frac{r\left(r-1\right)}{2}\alpha . \end{array}$$

Finding the maximum value of $\sum_{i<j}^{r}h_{i}h_{j}x^{T}(G_{ij}^{T}P+PG_{ij})\tilde{x}$ is equivalent to determining the maximum value of ∑_*i*<*j*_^*r*^ *h*_*i*_ *h*_*j*_ *λ*_max⁡_(*G*_*ij*_^*T*^ *P* + *PG*_*ij*_). This can be presented as a nonlinear programming. The optimal algorithms are employed to seek the best solution. Moreover, the MATLAB Optimization Toolbox consists of functions that minimize or maximize general nonlinear functions. By using the toolbox, the nonlinear programming is expressed in the following form:

(22) max⁡i,j    ∑i<jrhihjλmax⁡(GijTP+PGij) 1≤i<j≤rSubject  to ∑i=1rμi=1  μi≥0 ∑j=1rμj=1 μj≥0.

The largest eigenvalue of (*G*_*ij*_^*T*^ *P* + *PG*_*ij*_) can be obtained in advance, so the maximum value is determined to be

(23) $$\begin{array}{c} k_{2}=\underset{i,j}{\max}\sum_{i<j}h_{i}h_{j}\lambda_{\max}\left(G_{ij}^{T}P+PG_{ij}\right). \end{array}$$

The following supervisory control is chosen:

(24) $$\begin{array}{c} u_{s}=\{\begin{array}{cc} -\frac{B^{T}Px}{{||{\tilde{x}}^{T}PB||}^{2}}k{||\tilde{x}||}^{2}, & \text{if}\;||{\tilde{x}}^{T}PB||\neq 0\\ 0 & \text{if}\;||{\tilde{x}}^{T}PB||=0, \end{array} \end{array}$$

where *k* > *k*_*j*_, *j* = 1 or 2. If $||{\tilde{x}}^{T}PB||\neq 0$, then substituting (24) into (18) gives

(25) V˙1(x)≤−∑i=1rhi2x~TQix~+2kj||x~||2−2k||x~||2≤−∑i=1rhi2x~TQix~=−V2(x),

where *V*_2_(*x*) is a positive definite function. When $||{\tilde{x}}^{T}PB||=0$ can give the following form:

(26) x~T[(Ai−BiKi)TP+P(Ai−BiKi)]x  =x~T(AiTP+PAi)x~≤−x~TQix~, i=1,…,r,∑i<jrhihjx~T(GijTP+PGij)x~  =∑i<jrhihj[x~T(AiP−PAi)x~+x~T(AjTP−PAj)x~]  ≤−∑i<jhihjx~T(Qi+Qf)x~, 1≤i<j≤r,

the time derivative of *V*_1_(*x*) becomes

(27) $$\begin{array}{c} {\dot{V}}_{1}\left(x\right)\leq -\sum_{i=1}^{r}h_{i}^{2}{\tilde{x}}^{T}Q_{i}\tilde{x}-\sum_{i<j}h_{i}h_{j}x^{T}\left(Q_{i}+Q_{f}\right)\tilde{x}=-V_{3}\left(x\right), \end{array}$$

where *V*_3_(*x*) is a positive definite function. Thus, the closed-loop fuzzy system is asymptotically stable.

## 3. System Descriptions

**Figure 1:**
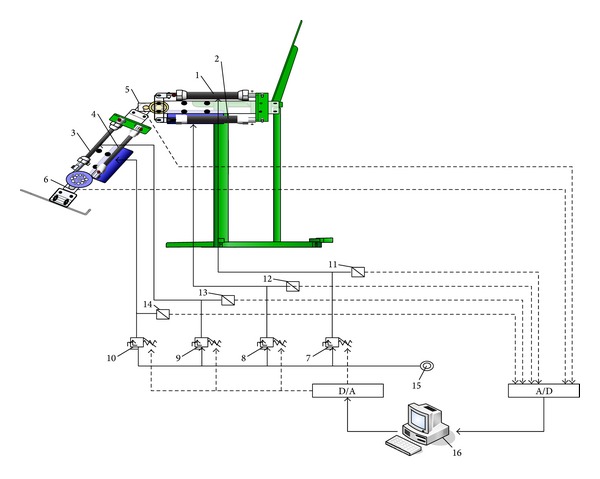
The experimental setup.

Figure 1 shows the experimental setup, including four PAMs, two rotary potentiometers, four pressure proportional valves, and four pressure transducers. The hardware includes an IBM-compatible personal computer to calculate the control signal, which controls the pressure proportional valve through a D/A card. The angles of the joints are detected using rotary potentiometers, the air pressure of each PAM is measured using pressure transducers, and the measurements are then fed back to the computer through an A/D card. These specifications are listed in Table 1.

**Figure 2:**
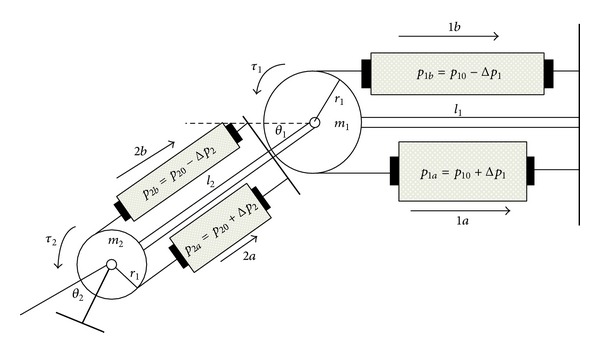
Operation principle of the leg rehabilitation machine driven by PAMs.

Figure 2 presents the operation principle of the leg rehabilitation machine, depicting a two-joint leg. The behavior of the leg manipulated by the rehabilitation machine is similar to that of a human leg. Output angles *θ*_1_ and *θ*_2_ simulate the knee and ankle joints, and the ranges of the rotary angles *θ*_1_ and *θ*_2_ are from −45° to 45° and from −50° to 50°, respectively. The link mass *m*_1_ = 2.7 kg, *m*_2_ = 0.81 kg, and the link length *l*_1_ = 0.5 m, *l*_2_ = 0.26 m. The rotating torque *τ* is generated by the difference in pressure Δ*p* between the two opposing PAMs. That is, when *p*_*a*_ > *p*_*b*_, as in Figure 2, the torque exerted on the joint is counterclockwise and the rotation of the joint is also counterclockwise.

So, a pair of such PAMs is tied together around a pulley with a radius *r*_*i*_, as in Figure 2. Then, the torque values imparted to the pulley by the PAM pair are [12]

(28) $$\begin{array}{c} \tau_{1}=\left(\phi_{1a}-\phi_{1b}\right)r_{1}\\ \tau_{2}=\left(\phi_{2a}-\phi_{2b}\right)r_{2}, \end{array}$$

where

(29a) $$\begin{array}{c} \phi_{1a}=F_{1a}\left(p_{1a}\right)-K_{1a}\left(p_{1a}\right)r_{1}\theta_{1}-B_{1a}\left(p_{1a}\right)r_{1}{\dot{\theta }}_{1} \end{array}$$

(29b) $$\begin{array}{c} \phi_{1b}=F_{1b}\left(p_{1b}\right)-K_{1b}\left(p_{1b}\right)r_{1}\theta_{1}-B_{1b}\left(p_{1b}\right)r_{1}{\dot{\theta }}_{1} \end{array}$$

(29c) $$\begin{array}{c} \phi_{2a}=F_{2a}\left(p_{2a}\right)-K_{2a}\left(p_{2a}\right)r_{2}\theta_{2}-B_{2a}\left(p_{2a}\right)r_{2}{\dot{\theta }}_{2} \end{array}$$

(29d) $$\begin{array}{c} \phi_{2b}=F_{2b}\left(p_{2b}\right)-K_{2b}\left(p_{2b}\right)r_{2}\theta_{2}-B_{2b}\left(p_{2b}\right)r_{2}{\dot{\theta }}_{2}, \end{array}$$

where the spring coefficient *K*(*p*) and the damping coefficient *B*(*p*) are given by Reynolds et al. [13].

The desired input pressures $P_{a}={\left[\begin{array}{cc} p_{1a} & p_{2a} \end{array}\right]}^{T}$ and $P_{b}={\left[\begin{array}{cc} p_{1b} & p_{2b} \end{array}\right]}^{T}$ for each PAM are generated by the following equation:

(30) $$\begin{array}{c} P_{a}\left(t\right)=P_{0}+\Delta P\left(t\right),\;\;P_{b}\left(t\right)=P_{0}-\Delta P\left(t\right), \end{array}$$

where $P_{0}={\left[\begin{array}{cc} p_{10} & p_{20} \end{array}\right]}^{T}$ is a nominal constant input PAM pressure and $\Delta P(t)={\left[\begin{array}{cc} \Delta p_{1} & \Delta p_{2} \end{array}\right]}^{T}$ is the control pressure input with an arbitrary function of time. Because the pressure input $\Delta P(t)={\left[\begin{array}{cc} \Delta p_{1} & \Delta p_{2} \end{array}\right]}^{T}$ and output $\theta ={\left[\begin{array}{cc} \theta_{1} & \theta_{2} \end{array}\right]}^{T}$, the system can be written as a two-input, two-output (TITO) control system. The control signal $u={\left[\begin{array}{cc} u_{1} & u_{2} \end{array}\right]}^{T}$ is proportional to Δ**P** based on the pressure proportional valve's characteristics. That is, Δ**P** can be used instead of **u** as a control input.

## 4. Dynamic Model of a Two-Joint Leg Rehabilitation Machine Driven by PAMs

Figure 2 shows a two-joint leg rehabilitation machine driven by PAMs, and the dynamic equation is given as follows [14]:

(31) $$\begin{array}{c} M\left(\theta \right)\ddot{\theta }+C\left(\theta ,\dot{\theta }\right)\dot{\theta }+G\left(\theta \right)=\tau , \end{array}$$

where

(32) $$\begin{array}{c} M\left(\theta \right)=\left[\begin{array}{cc} \left(m_{1}+m_{2}\right)l^{2} & m_{2}l_{1}l_{2}\left(s_{1}s_{2}+c_{1}c_{2}\right)\\ m_{2}l_{1}l_{2}\left(s_{1}s_{2}+c_{1}c_{2}\right) & m_{2}l_{2}^{2} \end{array}\right]\\ C\left(\theta ,\dot{\theta }\right)=\left[\begin{array}{cc} 0 & -m_{2}l_{1}l_{2}\left(c_{1}s_{2}-s_{1}c_{2}\right){\dot{\theta }}_{2}\\ -m_{2}l_{1}l_{2}\left(c_{1}s_{2}-s_{1}c_{2}\right){\dot{\theta }}_{1} & 0 \end{array}\right]\\ G\left(\theta \right)=\left[\begin{array}{c} \left(m_{1}+m_{2}\right)l_{1}gs_{1}\\ -m_{2}l_{2}gs_{2} \end{array}\right] \end{array}$$

and *M*(*θ*) is the moment of inertia, $C\left(\theta ,\dot{\theta }\right)$ includes Coriolis and centripetal force, and *G*(*θ*) is the gravitational force. Notation *s*_1_ = sin(*θ*_1_), *s*_2_ = sin(*θ*_2_), *c*_1_ = cos⁡(*θ*_1_), and *c*_2_ = cos⁡(*θ*_2_). Let *x*_1_ = *θ*_1_, $x_{2}={\dot{\theta }}_{1}$, *x*_3_ = *θ*_2_, and $x_{4}={\dot{\theta }}_{2}$; then (31) can be written as the following state-space form [14]:

(33) $$\begin{array}{c} {\dot{x}}_{1}=x_{2}\\ {\dot{x}}_{2}=f_{1}\left(x\right)+g_{11}\left(x\right)\tau_{1}+g_{12}\tau_{2}\\ {\dot{x}}_{3}=x_{4}\\ {\dot{x}}_{4}=f_{2}\left(x\right)+g_{21}\left(x\right)+g_{22}\tau_{2}, \end{array}$$

where

(34) f1(x)=(s1c2−c1s2)[m2l1l2(s1s2+c1c2)x22−m2l22x42]l1l2[(m1+m2)−m2(s1s2+c1c2)2] +[(m1−m2)l2gs1−m2l2gs2(s1s2+c1c2)]l1l2[(m1+m2)−m2(s1s2+c1c2)2]f2(x)=(s1c2−c1s2)[−(m1+m2)l12x22+m2l1l2(s1s2+c1c2)x42]l1l2[(m1+m2)−m2(s1s2+c1c2)2] +[−(m1+m2)l1gs1(s1s2+c1c2)+(m1+m2)l1gs2]l1l2[(m1+m2)−m2(s1s2+c1c2)2]g11(x)=m2l22m2l12l22[(m1+m2)−m2(s1s2+c1c2)2]g12(x)=−m2l1l2(s1s2+c1c2)m2l12l22[(m1+m2)−m2(s1s2+c1c2)2]g21(x)=−m2l1l2(s1s2+c1c2)m2l12l22[(m1+m2)−m2(s1s2+c1c2)2]g22(x)=(m1+m2)l12m2l12l22[(m1+m2)−m2(s1s2+c1c2)2].

## 5. Experimental Studies

**Figure 3:**
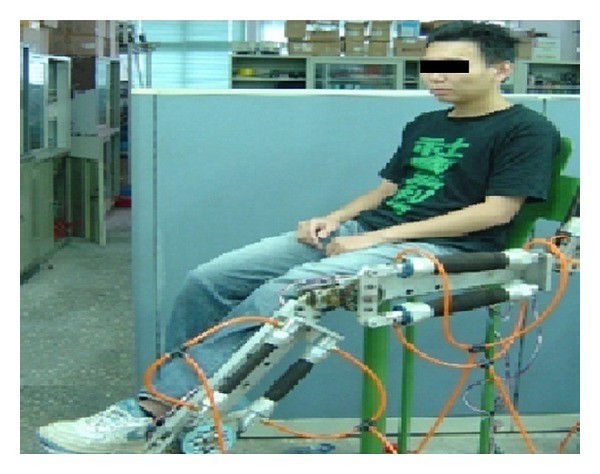
The two-joint leg rehabilitation device with actual human loading.

Figure 3 shows the leg rehabilitation machine using an actual human loading with a 65 kg weight. The automatic device can help patients to recover lower limb motion function by means of continuous passive motion, such as a sinusoidal wave command, an irregular curve command, and an end-effect tracking command. The experiments include both the proposed approach and PDC for comparison in order to evaluate efficacy and control performance. The controllers were implemented on an Intel Pentium 1.8 GHz PC with a sampling time of 5 ms, and the entire control software was coded in C++.

This study attempts to use as few rules as possible in order to minimize design effort and complexity. The T-S fuzzy model of the system is thus given the following four-rule fuzzy model:

(35) R1:IF  x1  is  about  (π/4)  and  x3  is  about  (π/4)THEN  x˙=A1x1+B1uR2:IF  x1  is  about  (π/4)  and  x3  is  about  (−π/4)THEN  x˙=A2x1+B2uR3:IF  x1  is  about  (−π/4)  and  x3  is  about  (π/4)THEN  x˙=A3x1+B3uR4:IF  x1  is  about  (−π/4)  and  x3  is  about  (−π/4)THEN  x˙=A4x1+B4u,

where

(36) $$\begin{array}{c} A_{1}=\left[\begin{array}{cccc} 0 & 1 & 0 & 0\\ 4.4082 & 0.0059 & 0.6742 & -0.0002\\ 0 & 0 & 0 & 1\\ -1.4572 & 0.0002 & -3.9722 & 0.0002 \end{array}\right]\\ A_{2}=\left[\begin{array}{cccc} 0 & 1 & 0 & 0\\ 4.6734 & 0.0049 & 0.532 & 0.0001\\ 0 & 0 & 0 & 1\\ -1.2145 & -0.0001 & -3.563 & 0.0001 \end{array}\right]\\ A_{3}=\left[\begin{array}{cccc} 0 & 1 & 0 & 0\\ 5.4782 & 0.0021 & 0.6876 & -0.0002\\ 0 & 0 & 0 & 1\\ 1.4525 & 0.0002 & 4.6723 & 0.0002 \end{array}\right]\\ A_{4}=\left[\begin{array}{cccc} 0 & 1 & 0 & 0\\ 4.9821 & -0.0044 & 0.6543 & 0.0002\\ 0 & 0 & 0 & 1\\ -1.1034 & -0.0002 & 3.5631 & 0.0003 \end{array}\right]\\ B_{1}=B_{4}=\left[\begin{array}{cc} 0 & 0\\ 1.4811 & -2.849\\ 0 & 0\\ -2.849 & 23.7417 \end{array}\right]\\ B_{2}=B_{3}=\left[\begin{array}{cc} 0 & 0\\ 1.1965 & 1.0297\\ 0 & 0\\ 1.0297 & 19.1501 \end{array}\right]\\ P=\left[\begin{array}{cccc} 16.2602 & -0.6640 & 1.0734 & -0.0095\\ -0.6640 & 0.3007 & -0.3455 & 0.2871\\ 1.0734 & -0.3455 & 0.4730 & -0.3458\\ -0.0095 & 0.2871 & -0.3458 & 0.3658 \end{array}\right]\\ K_{1}=\left[\begin{array}{cccc} -0.4509 & 0.1745 & -0.7182 & 0.0527\\ -0.2807 & 0.0034 & -0.0471 & 0.2639 \end{array}\right]\\ K_{2}=\left[\begin{array}{cccc} -0.4619 & 0.1238 & -0.7812 & 0.0351\\ -0.2827 & 0.0068 & -0.0851 & 0.1401 \end{array}\right]\\ K_{3}=\left[\begin{array}{cccc} -0.5902 & 0.2132 & -0.8910 & 0.0531\\ -0.4107 & 0.0117 & -0.0730 & 0.2989 \end{array}\right]\\ K_{4}=\left[\begin{array}{cccc} -0.4891 & 0.2109 & -0.6734 & 0.04145\\ -0.3180 & 0.0085 & -0.0631 & 0.3893 \end{array}\right] \end{array}$$

which guarantee the stability condition (17). MATLAB Toolbox is used to obtain parameters as *k*_1_ = 0.0251 and *k*_2_ = 0.01539. For comparison with the proposed controller, the PDC feedback gains are designed to be

(37) $$\begin{array}{c} {\hat{K}}_{1}=\left[\begin{array}{cccc} -0.3293 & 0.1023 & -0.2538 & 0.02317\\ -0.1783 & 0.0021 & -0.02672 & 0.4732 \end{array}\right]\\ K_{2}=\left[\begin{array}{cccc} -0.4278 & 0.1845 & -0.2451 & 0.0731\\ -0.2185 & 0.0026 & -0.0752 & 0.0923 \end{array}\right]\\ K_{3}=\left[\begin{array}{cccc} -0.6013 & 0.3461 & -0.5482 & 0.0231\\ -0.4421 & 0.0093 & -0.0651 & 0.3529 \end{array}\right]\\ K_{4}=\left[\begin{array}{cccc} -0.3756 & 0.3150 & -0.5391 & 0.0421\\ -0.4250 & 0.0023 & -0.0531 & 0.3597 \end{array}\right]. \end{array}$$

### 5.1. Sinusoidal Wave Response

**Figure 4:**
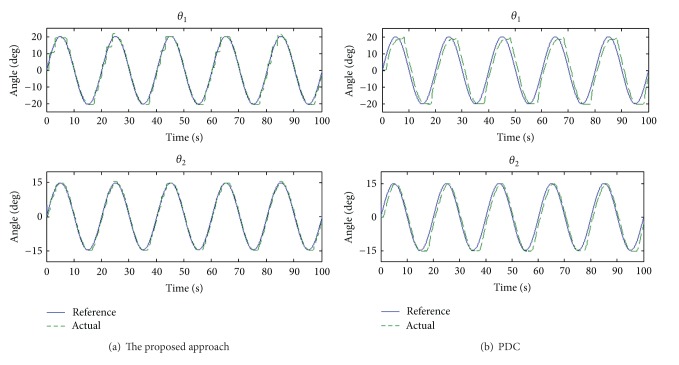
Sinusoidal wave response for knee and ankle joints.

**Figure 5:**
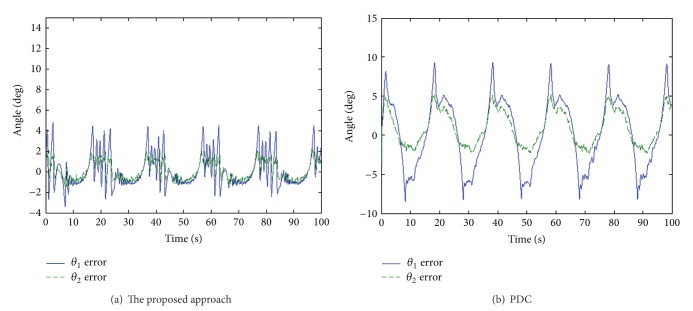
Angle tracking errors for both knee and ankle.

Continuous reciprocation is required in order to foster the recovery of extremity function. The sinusoidal wave responses of the proposed approach and PDC for both knee and ankle joints are shown in Figure 4. It is evident that angle trajectories of the proposed approach are close to the command.  Figure 5 shows that the proposed approach exhibits less tracking errors than does PDC. The peak-peak error and phase lag are listed in Table 2. Because of the interaction of the two joints, PDC has significant angle errors for *θ*_1_, which will degrade the rehabilitation effect. However, supervisory control can overcome the coupling effect of the two joints to achieve excellent rehabilitation function for patients.

### 5.2. Irregular Curve Response

In practical applications, it could be expected that the reference command will change with different input frequencies. The desired trajectories for both knee and ankle joints are

(38) $$\begin{array}{c} \theta_{1}=20*0.33\left(\sin\left(2\pi f_{1}t\right)+\sin\left(2\pi f_{2}t\right)+\sin\left(2\pi f_{3}t\right)\right)\\ \theta_{2}=15*0.33\left(\sin\left(2\pi f_{1}t\right)+\sin\left(2\pi f_{2}t\right)+\sin\left(2\pi f_{3}t\right)\right) \end{array}$$

with *f*_1_ = 0.05 Hz, *f*_2_ = 0.1 Hz, and *f*_3_ = 0.066 Hz.

**Figure 6:**
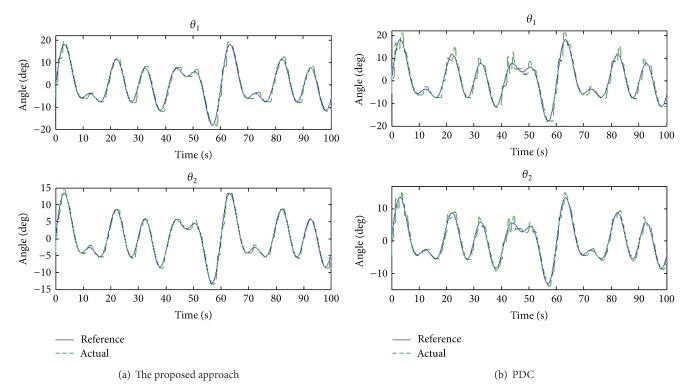
Irregular curve response for both knee and ankle joints.

**Figure 7:**
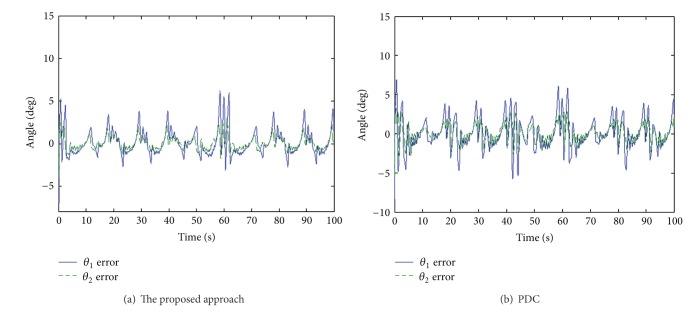
Angle tracking errors for both the proposed approach and PDC.

Figure 6 shows the tracking responses of irregular curves obtained using both the proposed approach and PDC. Tracking errors for the knee and ankle joints are shown in Figure 7. Clearly, the angle error of the proposed approach is average maintained within 2°. However, the proposed approach is capable of adapting to different frequencies.

### 5.3. Elliptic Response

The desired end-effect or trajectory is given by

(39) $$\begin{array}{c} x_{d}\left(t\right)=0.614-0.015\cdot \cos\left(0.2\pi \cdot t-\pi \right)\\ y_{d}\left(t\right)=-0.1\cdot \sin\left(0.2\pi \cdot t-2\pi \right), \end{array}$$

where 0 ≤ *t* ≤ 20 seconds.

**Figure 8:**
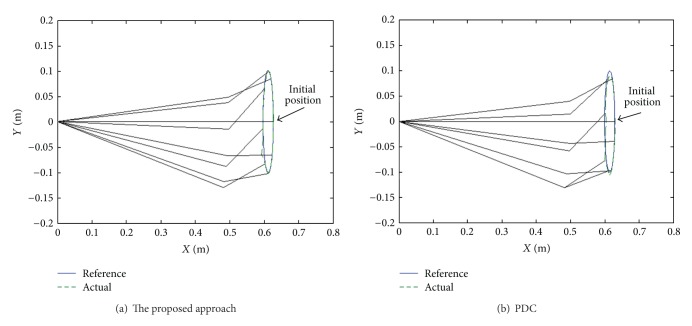
Elliptic response of the proposed approach and PDC.

**Figure 9:**
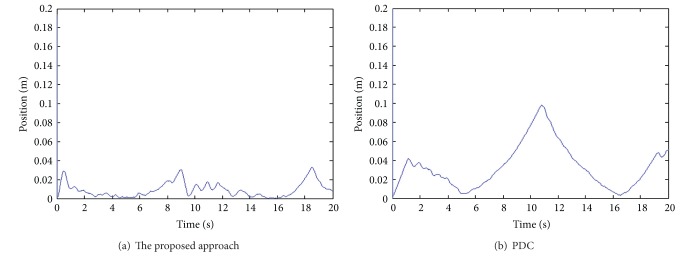
The end-effect position tracking errors.

**Figure 10:**
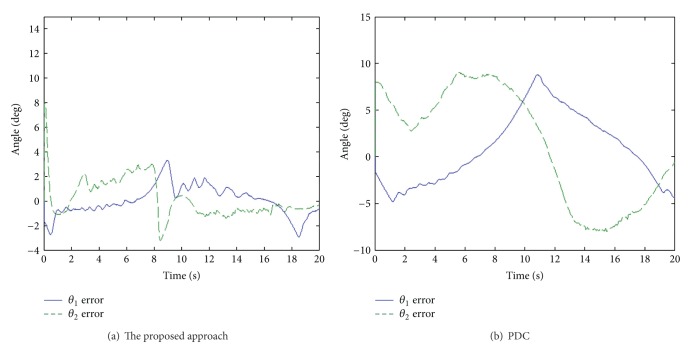
Angle tracking errors of knee and ankle joints.

The end-effect tracking responses in the *x*, *y* coordinate for both the proposed approach and PDC are shown in Figure 8, and the end-effect position tracking errors are displayed in Figure 9. It is evident that tracking behavior of the proposed approach is better than that of the PDC. As can be seen, the tracking errors of the proposed approach are within 0.03 m. On the other hand, angle tracking errors of knee and ankle joints are shown in Figure 10.

Moreover, it is difficult to enhance end-effector tracking performance using the PDC algorithm because the PDC cannot overcome the nonlinearity of PAMs and the structural interaction. However, the proposed approach overcomes successfully the coupling effect and parameter uncertainties of the system. As seen in the experimental results, the proposed approach can attain excellent end-effector tracking performance in rehabilitation function.

## 6. Conclusions

In this study, a novel composite fuzzy control is proposed and applied in the two-joint leg rehabilitation device driven by PAMs. The proposed controller is not only capable of decomposing nonlinear systems into a set of linear subsystems, but is also capable of simplifying a complex nonlinear system using linear control techniques, with the control gains determined using MATLAB's LMI Toolbox based on the Lyapunov stability theorem. Moreover, the supervisory control can overcome the coupling effect for a leg rehabilitation machine. Experimental results show that the system response of the proposed approach was in good agreement with that of the reference input and guarantee robustness to system parameter uncertainties.

## Figures and Tables

**Table 1:** Component specifications.

Number	Component	Specifications
1, 2, 3, 4	PAM	Festo, MAS-20-150N
7, 8, 9, 10	Pressure proportional valve	Mac, PPC5C
5, 6	Rotary potentiometer	Keen Engineering, KRT2050
11, 12, 13, 14	Pressure transducer	Jihsense, SN: 911166, 0–10 kgf/cm^2^
16	IBM-compatible PC	Pentium 1.8 GHz
	DAC, ADC	Automation, AIO3321

**Table 2:** Peak-peak error and phase lag for Figure 5.

The proposed approach				PDC			
Peak-peak error		Phase lag		Peak-peak error		Phase lag	
θ_1_	θ_2_	θ_1_	θ_2_	θ_1_	θ_2_	θ_1_	θ_2_
0.7%	0.35%	4.9°	4.5°	1.3%	0.65%	16.2°	10.8°
